# Interleukin-9 Deletion Relieves Vascular Dysfunction and Decreases Blood Pressure via the STAT3 Pathway in Angiotensin II-Treated Mice

**DOI:** 10.1155/2020/5741047

**Published:** 2020-02-14

**Authors:** Yunzhao Yang, Shaoqun Tang, Chunchun Zhai, Xin Zeng, Qingjian Liu, Cheng Xu, Hexiang Chen

**Affiliations:** Department of Anesthesiology, Wuhan University, Renmin Hospital, Wuhan, 430060 Hubei Province, China

## Abstract

**Background:**

Multiple interleukin (IL) family members were reported to be closely related to hypertension. We aimed to investigate whether IL-9 affects angiotensin II- (Ang II-) induced hypertension in mice.

**Methods:**

Mice were treated with Ang II, and IL-9 expression was determined. In addition, effects of IL-9 knockout (KO) on blood pressure were observed in Ang II-infused mice. To determine whether the effects of IL-9 on blood pressure was mediated by the signal transducer and activator of the transcription 3 (STAT3) pathway, Ang II-treated mice were given S31-201. Furthermore, circulating IL-9 levels in patients with hypertension were measured.

**Results:**

Ang II treatment increased serum and aortic IL-9 expression in a dose-dependent manner; IL-9 levels were the highest in the second week and continued to remain high into the fourth week after the treatment. IL-9 KO downregulated proinflammatory cytokine expression, whereas it upregulated anti-inflammatory cytokine levels, relieved vascular dysfunction, and decreased blood pressure in Ang II-infused mice. IL-9 also reduced smooth muscle 22*α* (SM22*α* (SM22

**Conclusions:**

IL-9 KO alleviates inflammatory response, prevents phenotypic transformation of smooth muscle, reduces vascular dysfunction, and lowers blood pressure via the STAT3 pathway in Ang II-infused mice. IL-9 might be a novel target for the treatment and prevention of clinical hypertension.

## 1. Introduction

Hypertension is the most common disease in the world, and the total number of people with hypertension is estimated to exceed 1 billion globally [1]. The causes of hypertension are very complex, with many pathological factors contributing to it [2–4]. Among them, the inflammatory response of blood vessels is considered the most significant cause and has garnered increasing attention of researchers [2–4].

Interleukin (IL) is a multifunctional cytokine with a strong regulatory effect on inflammatory response. Increasing number of IL family members is found to be involved in the development of hypertension. Deletion of IL-6, IL-17, and IL-22 was reported to attenuate angiotensin II- (Ang II-) induced hypertension [5–10]. IL-17 treatment can even lead to hypertension without Ang II infusion in wild-type (WT) mice [11]. Knockout of IL-12p35, the subunit shared by IL-12 and IL-35, significantly elevated blood pressure in Ang II-infused mice; treatment with IL-12, a proinflammatory cytokine, rather than the anti-inflammatory cytokine IL-35, surprisingly reduced Ang II-induced hypertension in mice [12]. However, the effects of IL-10 on hypertension are still controversial, and both the anti- and the prohypertensive roles have been reported in previous studies [13, 14].

IL-9 belongs to the IL-2 superfamily and is mainly secreted by T helper 9 (Th9) cells in the inflammatory response. IL-9 binds to the IL-9 receptor (IL-9R) on target cells, activates the signal transducers and activators of the transcription (STAT3) pathway, and plays a proinflammatory role in various diseases [15]. IL-9 is critical for the progression of cardiovascular diseases as shown by several studies. Circulating Th9/IL-9 levels were increased in patients with acute coronary syndrome (ACS) and in atherosclerotic mice, whereas recombinant mouse IL-9 treatment aggravated the development of atherosclerosis in high-fat diet-fed ApoE-/- mice. Thus, IL-9 may be the novel contributing factor in atherosclerosis [16–18]. Recent studies also reported that Th9/IL-9 levels were increased in patients with aortic dissection and in myocardial infarction/reperfusion mice [19, 20]. In coxsackievirus B3-induced myocarditis, IL-9 reduces viral replication and thus reduces myocardial injury [21]. However, whether IL-9 affects Ang II-induced hypertension is still unknown. In the present study, IL-9 KO mice were used to investigate the effects of IL-9 on Ang II-induced hypertension and to explore possible underlying mechanisms.

## 2. Materials and Methods

### 2.1. Animals and Animal Models

IL-9 KO mice and WT mice with a C57BL/6 background were purchased from the Institute of Model Zoology of Nanjing University (China) and housed in a specific-pathogen-free mouse room in the Renmin Hospital of Wuhan University. Mice aged 10 weeks were used for this study. First, WT mice were administered Ang II (750 ng/kg/min, Enzo) for different time periods (1 week, 2 weeks, or 4 weeks) or infused with different doses of Ang II (250 ng/kg/min, 500 ng/kg/min, or 750 ng/kg/min) for 4 weeks; mice in the control group received saline (*n* = 6 in each group). In addition, both WT mice and IL-9 KO mice were infused with Ang II (750 ng/kg/min) for 4 weeks (*n* = 10 in each group). At the end of chronic infusion, blood and aortas were harvested for further analysis. Furthermore, Ang II-infused WT mice and IL-9 KO mice were, respectively, daily treated with vehicle (50 *μ*l, DMSO) and/or S31-201 (2.5 mg/kg, a special STAT3 inhibitor) (*n* = 10 for each group) [10]. This study was reviewed and approved by the Institutional Animal Care of Renmin Hospital of Wuhan University (2017RM0411M).

### 2.2. Establishment of a Mouse Hypertension Model

Osmotic minipumps were incubated in a water bath for 72 hours at 37°C according to the manufacturer's instructions. Then, Ang II was diluted with saline and injected into osmotic minipumps. Mice were anesthetized with 2% isoflurane, the skin of the neck was cut open, and the osmotic minipumps were implanted in the skin of the neck. Finally, the skin was stitched together.

### 2.3. Detection of Aortic Protein Expression

Total protein was obtained from aortic tissue and quantified. Total protein was separated and transferred onto Polyvinylidene Fluoride (PVDF) membranes (Millipore). Blots were then blocked with nonfat milk and then incubated with anti-IL-9, anti-IL-9R (both from GeneTex), anti-GAPDH, anti-total-STAT3 (T-STAT3), anti-phosphorylation-STAT3 (P-STAT3, triple from Cell Signaling Technology), anti-OPN, and anti-SM22*α* (both form Abcam) antibodies overnight at 4°C. Subsequently, the blots were incubated with the secondary antibody and scanned using Odyssey.

### 2.4. Measurement of Serum Cytokine Levels

Serum was collected from blood samples by centrifugation. Levels of IL-9, IL-1*β*, IL-6, IL-17, tumor necrosis factor-*α* (TNF-*α*), interferon-*γ* (IFN-*γ*), monocyte chemoattractant protein-1 (MCP-1), IL-4, IL-10, and IL-13 in mice and IL-9 in human were detected using ELISA kits (all from eBioscience) according to the manufacturer's instructions. All samples were analyzed twice.

### 2.5. Blood Pressure Measurement

Two methods were used to measure blood pressure. First, mice were placed in a fixator and then on a heating plate to warm them; the pressurized tail sleeve was then placed on the tail of each mouse, and the program was started. Data for systolic blood pressure (SBP) before and after Ang II infusion were obtained. At the end of Ang II infusion, mice were anesthetized and a microtip catheter transducer (Millar, Inc. A) was inserted into the right carotid artery, and SBP, diastolic blood pressure (DBP), mean arterial pressure (MAP), and heart rate (HR) data were collected using a Millar Pressure Volume System (Millar, Inc.).

### 2.6. Detection of Vascular Function

Fresh aortas were dissected; the surrounding tissue was removed and then immediately placed in cold physiological salt solution as described in a previous study [22]. Aortas were cut into 3-4 mm aortic rings, connected to the isometric force transducer, and transferred to a container with physiological salt solution at 37°C and 95% O_2_ and 5% CO_2_ bubbling. Concentration-force curves in response to acetylcholine (ACh) and sodium nitroprusside (SNP) stimulation following contraction to an EC70 concentration (1 mmol/L) of phenylephrine (PE) were obtained in a half-log, cumulative fashion. Parts of the aortic rings were treated with indomethacin (Indo, 10 *μ*mol/L, 60 min) before the measurement.

### 2.7. Analysis of mRNA Expression

Total mRNA was isolated from aortic tissue and vascular smooth muscle cells (SMCs) using TRIzol reagent. cDNA was synthesized from RNA samples using a reverse transcription kit. LightCycler 480 SYBR Green Master Mix was used to perform PCR amplification for the detection of target genes, including IL-9, IL-9R, IL-1*β*, IL-6, IL-17, TNF-*α*, IFN-*γ*, MCP-1, IL-4, IL-10, IL-13, OPN, and SM22*α*. Gene expression levels were normalized against GAPDH expression. Primer sequences used for RT-qPCR are shown in Table 1. All the reagents used for this experiment were purchased from Roche (Germany).

### 2.8. Cell Culture and Detection

Mouse aortic SMCs were purchased from the National Infrastructure of Cell Line Resource (China) and cultured in the Roswell Park Memorial Institute (RPMI) 1640 complete culture medium; 1% penicillin-streptomycin was also added to the culture medium. Cultures were incubated at 37°C in a humidified atmosphere with 5% CO_2_. After starvation for 12 hours, mouse SMCs were treated with saline, recombinant mouse IL-9 (rIL-9, 50 ng/mL, PeproTech), S31-201 (10 *μ*M, Sigma), and/or Ang II (100 nmol/l) [23, 24]. After treatment for 24 hours, total RNA was isolated from the SMCs for further analysis.

### 2.9. Human Blood Sample Collection and Serum IL-9 Analysis

Blood samples were collected from patients with hypertension (*n* = 80) and control subjects (*n* = 50) from the Renmin Hospital of Wuhan University from September 2017 to May 2018. Serum was collected from each blood sample, and serum IL-9 levels in each sample were detected using human IL-9 ELISA kits according to the manufacturer's instructions. All blood specimen donors or their family members signed informed consent forms. This study was approved by the ethics committee of the Renmin Hospital of Wuhan University. Clinical data of patients with hypertension and control subjects are listed in Table 2.

### 2.10. Data Analysis

Data from animal studies were expressed as the mean ± standard deviation (SD); differences between two groups and more than two groups were compared using Student's *t*-test and one-way or two-way ANOVA followed by Tukey's multiple comparisons test, respectively. Clinical characteristics are presented as the median (lower quartile to upper quartile) or counts (percentages) and compared using the Mann-Whitney *U* test or the chi-square test. Spearman's correlation analysis was used to evaluate correlations between blood pressure and circulating IL-9 levels in patients with hypertension. Multiple ANOVA (type III sums of squares) was performed to investigate the association between plasma IL-9 levels and individual risk factors in human subjects. All data were analyzed using the SPSS 23.0 software, and *p* < 0.05 was considered to be statistically significant.

## 3. Results

### 3.1. Chronic Ang II Infusion Elevated IL-9 Expression in Mice

**Figure 1:**
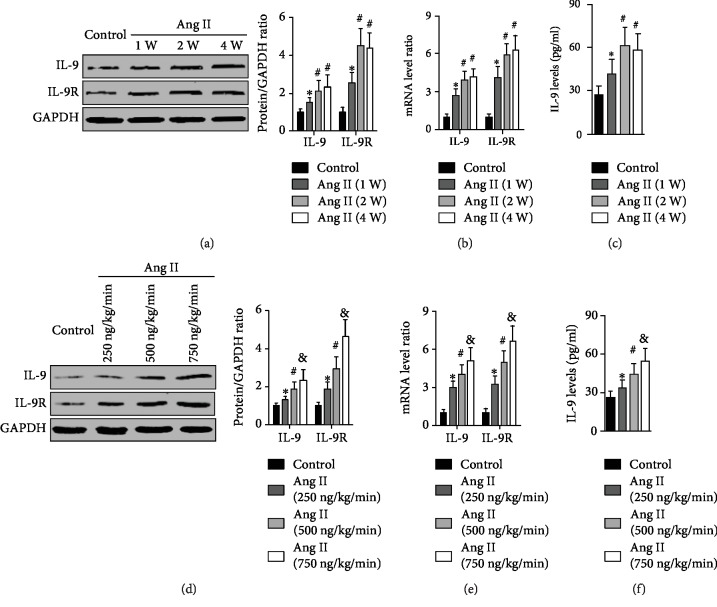
Effects of Ang II treatment on IL-9 expression. (a) Aortic IL-9 and IL-9R expressions were measured at different time points of Ang II infusion. (b) Aortic mRNA expression of IL-9 and IL-9R of each group was determined. (c) Serum IL-9 levels were detected at each time point. (d) Aortic IL-9 and IL-9R expressions were investigated at different doses of Ang II at the end of four weeks. (e) IL-9 mRNA and IL-9R mRNA in aortas of mice treated with different concentrations of Ang II were detected. (f) Serum IL-9 levels were determined when different Ang II doses were administered. *N* = 5‐6 for each group. ^∗^*p* < 0.05 vs. the control group; ^#^*p* < 0.05 vs. the Ang II (1 W) group or the Ang II (250 ng/kg/mini) group; ^&^*p* < 0.05 vs. the Ang II (500 ng/kg/min) group.

As compared with the control group, the expression of protein and mRNA of both IL-9 and IL-9R in aortic samples was significantly increased by week 1 after initiation of Ang II infusion, continued to increase and reached a maximum by week 2, and was maintained at high levels up to the end of week 4 (Figures 1(a) and 1(b)). Similar trends were observed for serum IL-9 levels (Figure 1(c)). In addition, Ang II treatment increased aortic IL-9 and IL-9R expression, as well as serum IL-9 levels, in a dose-dependent manner (Figures 1(d)–1(f)).

### 3.2. IL-9 KO Reversed Blood Pressure in Ang II-Infused Mice

**Figure 2:**
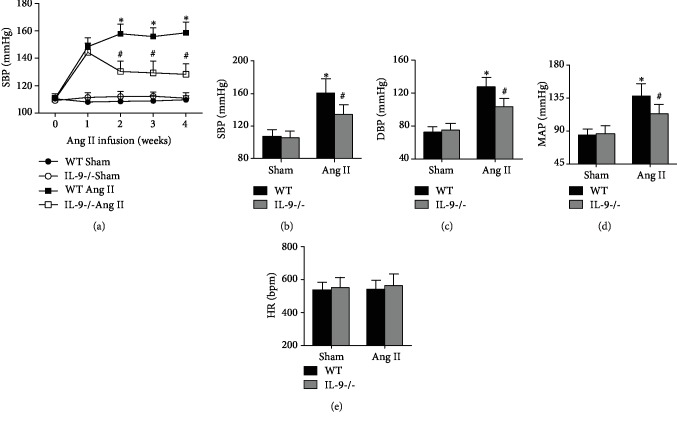
Effects of IL-9 KO on Ang II-induced hypertension. (a) Systolic blood pressure (SBP) of saline and angiotensin II-infused mice was measured using the tail-cuff method. (b–d) SBP, diastolic BP, mean arterial pressure, and heart rate were detected using the Millar Pressure Volume System. *N* = 10 for each group; ^∗^*p* < 0.05 vs. the WT Sham group; ^#^*p* < 0.05 vs. the WT Ang II group.

Results of the noninvasive tail cuff method showed no differences in SBP among the four groups before Ang II infusion (Figure 2(a)). The SBP in Ang II-infused mice began rising in the first week, reached a maximum value in the second week, and remained at the maximum value until the fourth week (Figure 2(a)). IL-9 KO had no significant effect on SBP during the first week of Ang II infusion; however, SBP significantly decreased in IL-9 KO mice from the second week to the fourth week (Figure 2(a)). Results of internal carotid artery invasive measurement showed that the SBP, DBP, and MAP decreased significantly after Ang II infusion in IL-9 KO mice (Figures 2(b)–2(d)). Meanwhile, both Ang II-infusion and IL-9 KO did not affect the HR (Figure 2(e)).

### 3.3. IL-9 KO Alleviated Ang II-Induced Vascular Dysfunction

**Figure 3:**
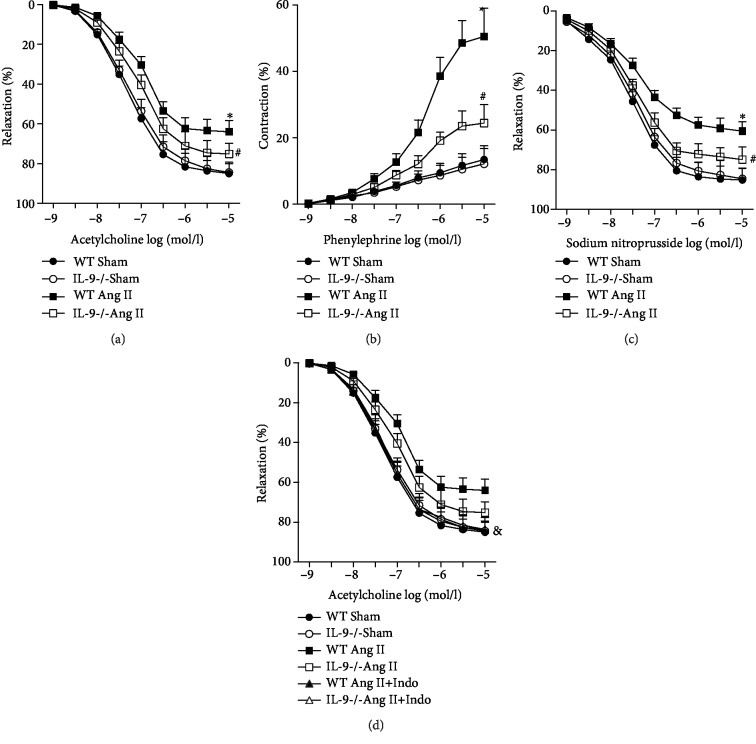
The regulatory effect of IL-9 KO on vascular dysfunction. (a) The effect of IL-9 KO on ACh-induced relaxation was measured in endothelial-intact aortas isolated from the four groups. (b) The effect of IL-9 KO on PE-induced contraction was measured in endothelial-intact aortas isolated from each group. (c) The effect of IL-9 KO on SNP-induced relaxation was measured in endothelial-intact aortas isolated from each group. (d) Effect of indomethacin on ACh-induced relaxation. *N* = 5 in each group. ^∗^*p* < 0.05 vs. the WT Sham group; ^#^*p* < 0.05 vs. the WT Ang II group; ^&^*p* < 0.05 vs. the IL-9-/- Ang II group.

After 4 weeks of Ang II infusion, endothelium-intact aortas were dissected in order to investigate vascular reactivity, including endothelium-dependent and endothelium-independent relaxation responses and contraction responses. ACh-induced endothelium-dependent relaxation and SNP-induced endothelium-independent relaxation were significantly reduced by Ang II treatment, whereas PE-induced contraction was elevated upon Ang II treatment (Figures 3(a)–3(c)). IL-9 KO alleviated Ang II-induced relaxation and contraction of the vascular rings (Figures 3(a)–3(c)). Parts of the aortic rings were treated with indomethacin to determine whether the effect of IL-9 KO on vascular reactivity was mediated by inflammatory response. Indomethacin attenuated the effect of IL-9 KO on Ang II-induced vascular dysfunction (Figure 3(d)).

### 3.4. IL-9 KO Alleviated Both Systemic and Local Inflammation in Ang II-Treated Mice

**Figure 4:**
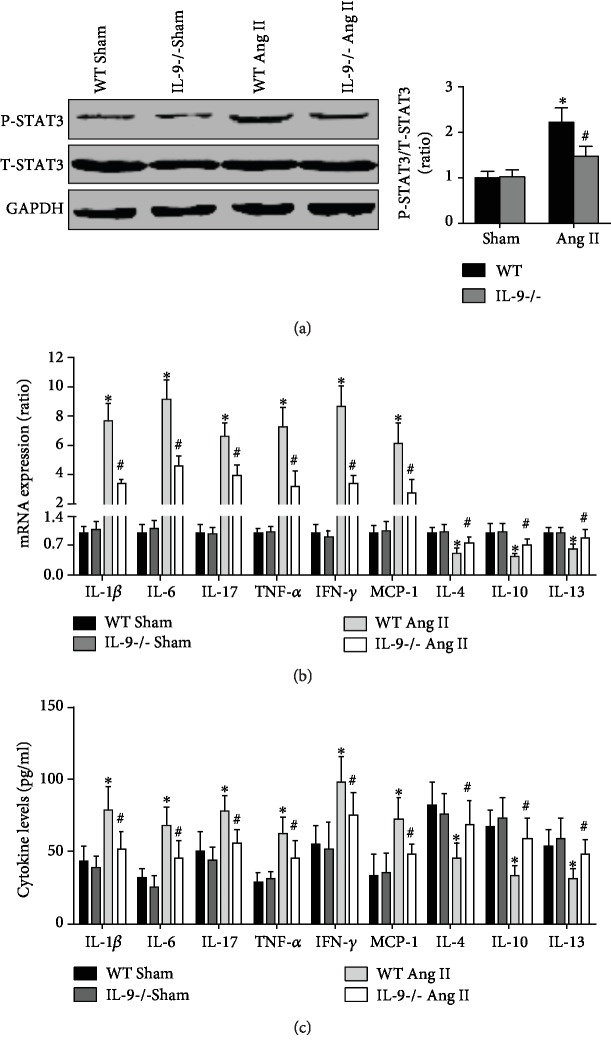
Effects of IL-9 KO on inflammatory response. (a) STAT3 phosphorylation for the four groups was measured. (b) The expression of aortic IL-1*β*, IL-6, IL-17, TNF-*α*, IFN-*γ*, MCP-1, IL-4, IL-10, and IL-13 mRNA in each group was analyzed. (c) Serum levels of these cytokines were detected using ELISA. *N* = 5 in each group. ^∗^*p* < 0.05 vs. the WT Sham group; ^#^*p* < 0.05 vs. the WT Ang II group.

First, we examined STAT3 activation. Ang II treatment significantly increased STAT3 phosphorylation, which was reversed upon IL-9 KO (Figure 4(a)). Next, mRNA expressions of various inflammatory cytokines in aortas were examined. IL-9 KO significantly increased IL-1*β*, IL-6, IL-17, TNF-*α*, IFN-*γ*, and MCP-1 mRNA levels, whereas it reduced IL-4, IL-10, and IL-13 mRNA expression in Ang II-treated mice (Figure 4(b)). In addition, similar trends were observed for the serum levels of these cytokines (Figure 4(c)).

### 3.5. IL-9 KO Reduced Ang II-Induced Phenotypic Transformation of Smooth Muscle

**Figure 5:**
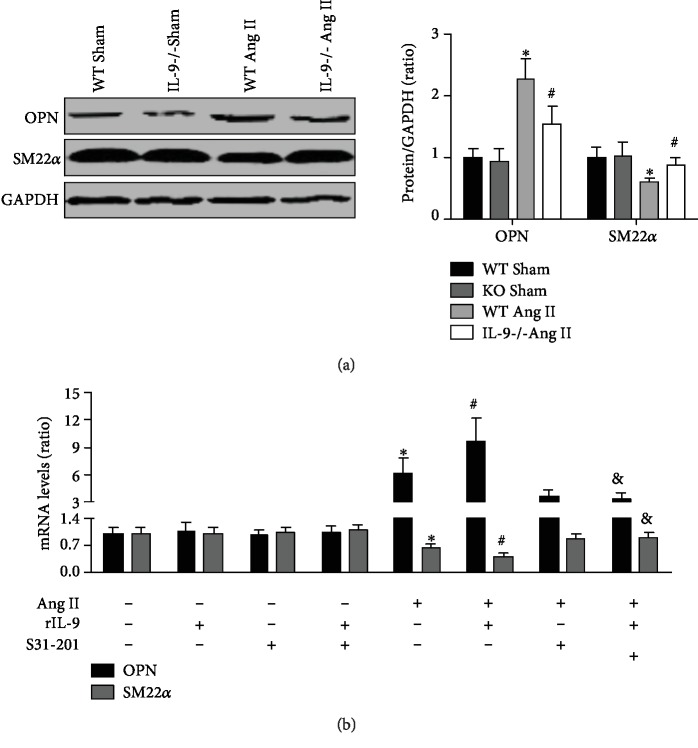
Effects of IL-9 KO on phenotypic transformation of smooth muscle. (a) Aortic OPN and SM22*α* expressions for the four groups were measured. *N* = 5 in each group. ^∗^*p* < 0.05 vs. the WT Sham group; ^#^*p* < 0.05 vs. the WT Ang II group. (b) Effects of rIL-9 and S31-201 on the OPN and SM22*α* mRNA expressions of Ang II-treated SMCs. *N* = 5 in each group. ^∗^*p* < 0.05 vs. the control group; ^#^*p* < 0.05 vs. the Ang II group; ^&^*p* < 0.05 vs. the Ang II+rIL-9 group.

The expression of OPN and SM22*α* in aortic samples was measured. Ang II treatment significantly increased OPN expression, whereas it decreased SM22*α* expression; these effects were prevented by IL-9 KO (Figure 5(a)). In vitro, OPN mRNA levels were increased in Ang II-treated SMCs, and they were further elevated when rIL-9 was added. These effects were significantly reversed by S31-201, a special inhibitor of the STAT3 pathway (Figure 5(b)). Opposite trends were observed for SM22*α* mRNA expression (Figure 5(b)).

### 3.6. Effects of IL-9 KO in Ang II-Treated Mice Were Mediated by S31-201

**Figure 6:**
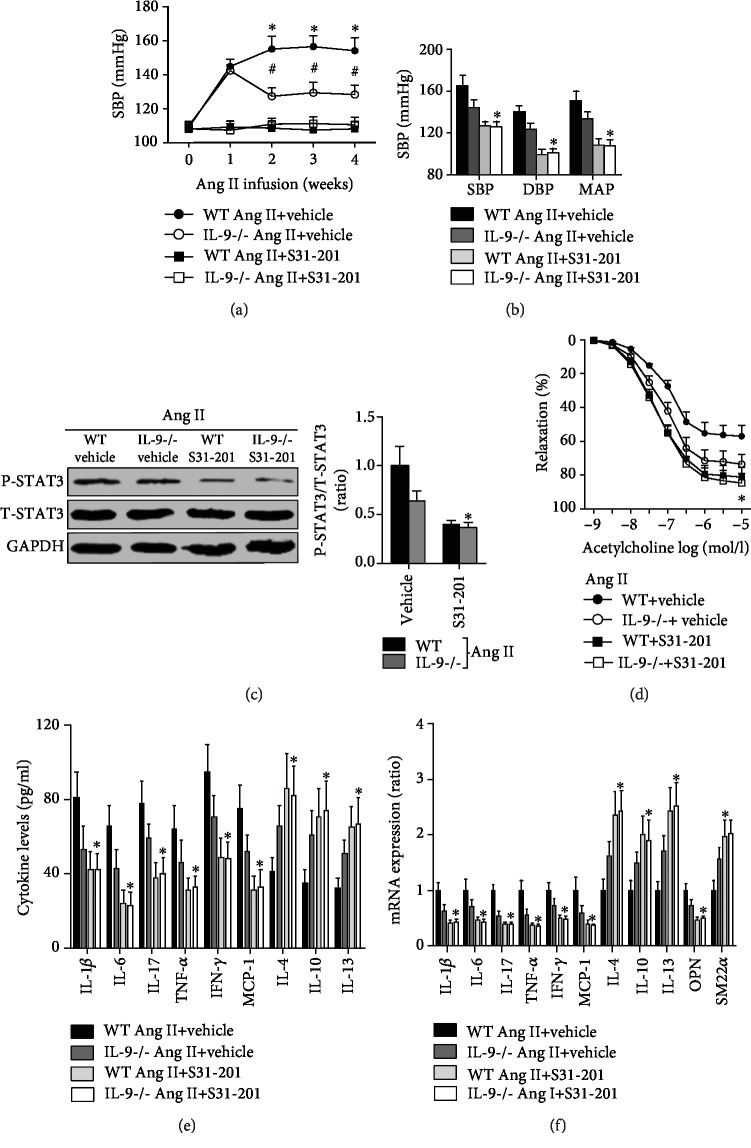
Effects of S31-201 on blood pressure, inflammation, and phenotypic transformation of smooth muscle. (a, b) Blood pressure was determined using the tail-cuff method and the Millar Pressure Volume System; *N* = 10 in each group. (c) The STAT3 phosphorylation in each group was measured. (d) The vascular function for the four groups was detected. (e) Serum cytokine levels were measured using ELISA kits. (f) Aortic mRNA expression of cytokines was analyzed by RT-PCR. *N* = 5 in each group; ^∗^*p* < 0.05 vs. the IL-9-/- Ang II+DMSO group.

S31-201 treatment significantly promoted the reduction of SBP, DBP, and MAP in both Ang II-treated WT mice and IL-9 KO mice, and the no differences of the blood pressure were observed between Ang II-infused WT mice and Ang II-infused IL-9 KO mice after S31-201 were given (Figures 6(a) and 6(b)). Treated with S31-201 also exhibited similar trends to STAT3 phosphorylation (Figure 6(c)). In addition, the regulatory role of IL-9 KO on Ang II-induced vascular dysfunction was further alleviated by S31-201 treatment (Figure 6(d)). S31-201 treatment could further decrease IL-1*β*, IL-6, IL-17, TNF-*α*, IFN-*γ*, and MCP-1 levels and mRNA expression, while increased IL-4, IL-10, and IL-13 levels and mRNA expression (Figures 6(e) and 6(f)). Finally, the aortic OPN mRNA expression was further decreased and SM22*α* was further decreased in S31-201-treated IL-9 KO mice (Figure 6(f)).

### 3.7. Circulating IL-9 Levels Were Elevated in Hypertension Patients

**Figure 7:**
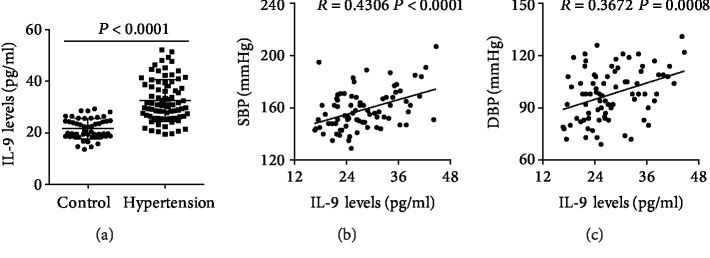
Serum IL-9 levels in patients with hypertension. (a) Serum IL-9 levels were measured using ELISA in patients with hypertension and control subjects. (b) Correlation analysis of SBP and IL-9 levels in patients with hypertension. (c) Correlation analysis of DBP and IL-9 levels in patients with hypertension. ^∗∗∗∗^*p* < 0.0001 vs. the control group.

Serum IL-9 levels were higher in patients with hypertension than in control subjects (Figure 7(a)). Spearman's correlation analysis showed that serum IL-9 levels positively correlated with both SBP and DBP (Figures 7(b) and 7(c)). In addition, multivariate analysis was performed to investigate the risk factors of hypertension that influence IL-9 secretion. Of the patients with hypertension, those with obesity and smoking habits exhibited higher IL-9 levels, whereas those with drinking habits and of male gender showed lower IL-9 levels. Other risk factors, including old age, hyperlipidemia, type 2 diabetes mellitus (T2DM), and family history, had no effect on IL-9 expression. Serum IL-9 levels, type III sum of square, *F* value, and *p* value for each group are listed in Table 3.

## 4. Discussion

In the present study, we found for the first time that IL-9 levels were significantly increased in both patients with hypertension and the Ang II-induced mouse model of hypertension. IL-9 KO significantly reduced inflammatory response, prevented vascular dysfunction, reversed phenotypic transformation of smooth muscle, and reduced blood pressured in Ang II-treated mice. In addition, the stimulatory effects of rIL-9 on phenotypic transformation of SMCs were reversed by S31-201 in vitro. S31-201 treatment further decreased the blood pressure, alleviated inflammation imbalance, and improved vascular dysfunction in Ang II-infused IL-9 KO mice. Our data showed that IL-9 can significantly regulate Ang II-induced hypertension, which might be mediated by the STAT3 pathway.

CD4+ T helper (Th) cells include regulatory T cells (Treg) and effector T cells, and the latter group includes Th1, Th2, and Th17 cells. Two novel subsets of effector T cells, Th22 and Th9, have also been discovered. These CD4+ Th cells are involved in responses to diseases via the secretion of characteristic inflammatory cytokines such as IFN-*γ*, IL-4, IL-9, IL-17, IL-22, and IL-35 for Th1, Th2, Th9, Th17, and Th22 cells, respectively [19]. Accumulating evidence has demonstrated that subsets of CD4+ Th cells are closely related to the development of hypertension and target organ damage. Among them, increased Th1 cell number and decreased Th2 cell number were observed in patients with hypertension. Exogenous Ang II treatment mediated a strong Th1 immune response and increased IFN-*γ* expression, whereas it reduced the Th2 immune response and decreased IL-4 secretion. Further, the changes in Th1/IFN-*γ* and Th2/IL-4 levels were related to Ang II-induced hypertension or the onset of kidney injury [25, 26]. These studies suggest that imbalance in Th1 and Th2 is closely related to the progression of hypertension or hypertensive complications. Ang II treatment also increased Th17 differentiation, whereas IL-17 deletion reversed hypertension in Ang II-treated mice [8]. A recent study reported that Ang II infusion significantly upregulated Th17 expression and downregulated Treg expression; the Th17/Treg imbalance decreased the inflammatory response and prevented target organ injury [27]. Th22/IL-22 levels were also increased in Ang II-treated mice. Exogenous IL-22 treatment aggravated endothelial dysfunction and elevated blood pressure, whereas an IL-22-neutralizing antibody had an exactly opposite effect [10]. This study also suggested that Th22 response plays a critical role in Ang II-induced hypertension.

IL-9 is a characteristic inflammatory cytokine, and the role of IL-9 in hypertension has not been reported. In the present study, we first examined the effect of Ang II infusion on IL-9 and IL-9R expressions, and we found that Ang II increased aortic IL-9 and IL-9R expressions. The results may suggest that IL-9 may participate in the process of Ang II-induced hypertension. Then, we further investigated the role of IL-9 KO on hypertension and IL-9 KO reduced blood pressure. Our results and those of other researchers have showed that hypertension is not caused or mediated by a specific type of immune cells or cytokines but by a combination of immune cells and cytokines. Therefore, multiple immune cells or their combined roles should be considered while designing treatment for hypertension or its complications.

The JAK-STAT pathway regulates a variety of biological effects, including apoptosis, oxidative stress, and especially inflammation [28]. IL-9 regulates the phosphorylation of STAT1/3/5. STAT3 is the primary IL-9 signaling pathway, and IL-9 participates in response to a variety of diseases by activating the STAT3 pathway [29]. Numerous studies have demonstrated that inhibition of the STAT3 pathway or deletion of suppressor of cytokine signaling 3 (SOCS3), which functions upstream of the STAT3 pathway, significantly reverses vascular dysfunction, reduces blood pressure elevation, and protects hypertension-related target organ injury [30, 31]. To investigate whether IL-9 is involved in hypertension through the regulation of inflammatory response, we detected STAT3 phosphorylation. Ang II-induced STAT3 activation was decreased upon IL-9 KO. We then examined the levels of inflammatory cytokines in systemic and in the aortas and found that IL-9 KO decreased proinflammatory cytokine mRNA expression, whereas it increased anti-inflammatory cytokine mRNA levels. In addition, the relieving role of IL-9 KO on vascular dysfunction was also completely reversed by indomethacin, a nonspecific anti-inflammatory drug. These results suggest that IL-9 might be involved in hypertension by regulating inflammatory response. However, the expression of various inflammatory factors was altered upon IL-9 KO, and indomethacin does not act only on certain inflammatory cytokines. Therefore, which inflammatory factor downstream of IL-9 plays a decisive role is still unclear and requires elucidation in subsequent studies. In addition, S31-201 treatment further improves the effects above by inhibiting the STAT3 pathway with s31-201, which can further confirm this hypothesis.

Normal vascular SMCs are mainly contractile, with a high expression of SM22*α* and low expression of OPN. Under the action of external factors, vascular SMCs convert to the secretory type, with reduced SM22*α* expression and increased OPN expression [12]. Systolic dysfunction caused by phenotypic transformation of smooth muscle is the fundamental cause of hypertension, and one of the important factors inducing its transformation is inflammatory response [12]. In the present study, we found that IL-9 KO reduced aortic OPN expression, whereas it increased SM22*α* levels. These results suggest that IL-9 KO partially reverses Ang II-induced phenotypic transformation of smooth muscle. Furthermore, to determine whether the effects of IL-9 on Ang II-induced phenotypic transformation of SMCs was mediated by the STAT3 signaling pathway, the Ang II-treated SMCs were also treated with recombinant mouse IL-9 and/or S31-201. Results showed that IL-9 treatment promoted phenotypic transformation of SMCs in vitro, and these effects could be reversed using a STAT3 inhibitor both in vitro and in vivo. These results further suggest that the regulatory effects of IL-9 on hypertension were mediated by the STAT3 pathway and achieved through the regulation of inflammatory response. In addition, results of clinical experiments showed that obesity and smoking, the risk factors for hypertension, could promote IL-9 expression. Considering that obesity and smoking were also closely related to inflammatory response, data of our clinical studies can also partly explain that IL-9 is involved in hypertension through inflammatory response.

In conclusion, our study demonstrated that IL-9 KO reduced blood pressure elevation by abating the inflammatory response and reversed the phenotypic transition of SMCs in Ang II-treated mice. Additionally, IL-9 KO plays a protective role in the Ang II-induced hypertension model. Thus, IL-9 might be a novel therapeutic agent for the prevention and treatment of clinical hypertension.

## Figures and Tables

**Table 1:** RT-PCR primers used.

Gene	Forward primer	Reverse primer
IL-9	AACAGTCCCTCCCTGTAGCA	AAGGATGATCCACCGTCAAA
IL-9R	TCCTGGTTCCTGATCTACAGC	TGTGTTTGATTTCAGTCACCTGG
IL-1*β*	GGGCCTCAAAGGAAAGAATC	TACCAGTTGGGGAACTCTGC
IL-6	AGTTGCCTTCTTGGGACTGA	TCCACGATTTCCCAGAGAAC
IL-17	TCCAGAAGGCCCTCAGACTA	AGCATCTTCTCGACCCTGAA
TNF-*α*	CCCAGGGACCTCTCTCTAATC	ATGGGCTACAGGCTTGTCACT
IFN-*γ*	ACTGGCAAAAGGATGGTGAC	TGAGCTCATTGAATGCTTGG
MCP-1	CTTCTGTGCCTGCTGCTCAT	CGGAGTTTGGGTTTGCTTGTC
IL-4	ACGAGGTCACAGGAGAAGGGA	AGCCCTACAGACGAGCTCACTC
IL-10	ATAACTGCACCCACTTCCCA	GGGCATCACTTCTACCAGGT
IL-13	CGCAAGGCCCCCACTAC	TGGCGAAACAGTTGCTTTGT
OPN	CGACGATGATGACGATGATGAT	CTGGCTTTGGAACTTGCTTGAC
SM22*α*	TCCAGTCCACAAACGACCAAGC	GAATTGAGCCACCTGTTCCATCTG
GAPDH	AACTTTGGCATTGTGGAAGG	CACATTGGGGGTAGGAACAC

**Table 2:** Clinical characteristics of patients with or without hypertension.

Characteristics	Control	Hypertension	*p* value
Elderly (*n*, %)	22 (44.0)	32 (40.0)	0.716
Male (*n*, %)	32 (64.0)	53 (66.3)	0.851
Smoked (*n*, %)	14 (28.0)	35 (43.8)	0.094
Drinking (*n*, %)	11 (22.0)	25 (31.3)	0.315
BMI > 25 (*n*, %)	29 (58.0)	61 (76.3)	0.033
Family history (*n*, %)	18 (36.0)	39 (48.8)	0.204
Hyperlipidemia (*n*, %)	11 (22.0)	24 (30.0)	0.417
T2DM (*n*, %)	1 (2.0)	3 (3.8)	0.999
Age (years)	57 (39, 71)	58 (43, 71)	0.712
BMI (kg/m^2^)	25.2 (23.8, 27.2)	25.7 (24.2, 27.9)	0.672
SBP (mmHg)	120 (109, 130)	164 (149, 173)	<0.001
DBP (mmHg)	75 (69, 83)	101 (89, 109)	<0.001
HR (bpm)	72 (63, 79)	74 (66, 81)	0.414
CREA (*μ*mol/L)	75 (66, 89)	75 (63, 85)	0.816
Glu (mmol/L)	5.3 (4.6, 5.7)	5.5 (4.8, 5.9)	0.398
TC (mmol/L)	4.6 (4.1, 5.1)	4.5 (4.0, 5.0)	0.664
TG (mmol/L)	1.4 (1.1, 2.1)	1.4 (1.1, 2.0)	0.782
HDL-C (mmol/L)	1.4 (0.9, 1.6)	1.3 (1.0, 1.6)	0.279
LDL-C (mmol/L)	2.4 (1.9, 2.9)	2.3 (1.8, 2.7)	0.198
Medications (*n*, %)			
ACEI/ARB	0 (0)	55 (68.8)	<0.001
*β*-Blockers	0 (0)	20 (25.0)	<0.001
CCB	0 (0)	60 (75.0)	<0.001
Diuretic	0 (0)	21 (26.3)	<0.001

BMI: body mass index; T2DM: type 2 diabetes mellitus; SBP: systolic blood pressure; DBP: diastolic blood pressure; HR: heart rate; CREA: creatinine; Glu: fasting glucose; TC: total cholesterol; TG: total triglycerides; HDL-C: high-density lipoprotein cholesterol; HDL-C: low-density lipoprotein cholesterol; ACEI: angiotensin-converting enzyme inhibitor; ARB: angiotensin receptor blocker; CCB: calcium channel blockers.

**Table 3:** Serum IL-9 levels in patients with concomitant risk factors for hypertension.

Factors	Number with factor	Number without factor	Serum IL-9 levels		Type III of square	*F* value	*p* value
			With factor	Without factor			
Hypertension	80	50	33.2 (24.3, 40.9)	24.1 (18.3, 27.9)	372316	178	<0.001
Hyperlipidemia	35	95	28.2 (21.3, 35.9)	27.9 (21.1, 35.2)	28	0.01	0.952
T2DM	4	126	27.7 (20.3, 34.7)	28.4 (21.6, 36.1)	122	1.09	0.468
Male	85	45	25.5 (21.4, 33.8)	30.7 (25.4, 38.7)	19523	104	0.008
Elderly	54	76	28.5 (24.9, 34.2)	28.9 (25.1, 35.1)	4523	29	0.774
Smoking	49	81	31.5 (25.4, 35.5)	27.5 (23.4, 31.6)	5263	54	0.002
Drinking	36	94	24.1 (20.7, 34.1)	31.7 (21.9, 39.9)	415321	264	<0.001
Obesity	90	40	29.9 (24.1, 36.1)	26.9 (23.3, 33.1)	872	32	0.028
Family history	57	73	28.7 (23.5, 35.1)	27.8 (22.9, 34.7)	6732	136	0.198

Hyperlipidemia: total plasma TC > 5.2 mmol/L or LDL‐C > 3.1 mmol/L; T2DM: according to the results of oral glucose tolerance test; elderly: age ≥ 60 years; smoking: current or within the last 6 months; drinking: more than 150 mL/day, current or within the last 6 months; obesity: BMI ≥ 25 kg/m^2^; family history: at least one person of the parents suffering hypertension.

## Data Availability

We declare that the materials described in the manuscript, including all relevant raw data, will be freely available to any scientist wishing to use them for noncommercial purposes, without breaching participant confidentiality.
