# Corrigendum to “Interleukin-9 Deletion Relieves Vascular Dysfunction and Decreases Blood Pressure via the STAT3 Pathway in Angiotensin II-Treated Mice”

**DOI:** 10.1155/2020/8537832

**Published:** 2020-10-30

**Authors:** Yunzhao Yang, Shaoqun Tang, Chunchun Zhai, Xin Zeng, Qingjian Liu, Cheng Xu, Hexiang Chen

**Affiliations:** Department of Anesthesiology, Wuhan University, Renmin Hospital, Wuhan, 430060 Hubei Province, China

In the article titled “Interleukin-9 Deletion Relieves Vascular Dysfunction and Decreases Blood Pressure via the STAT3 Pathway in Angiotensin II-Treated Mice” [1], the authors apologize that a blot of P-STAT3 which did not belong to this study was mistakenly used in (Figure 6(c)). The statistical results of P-STAT3 protein expression were correct, and the corrected (Figure 6(c)) is shown below.

## Figures and Tables

**Figure 1:**
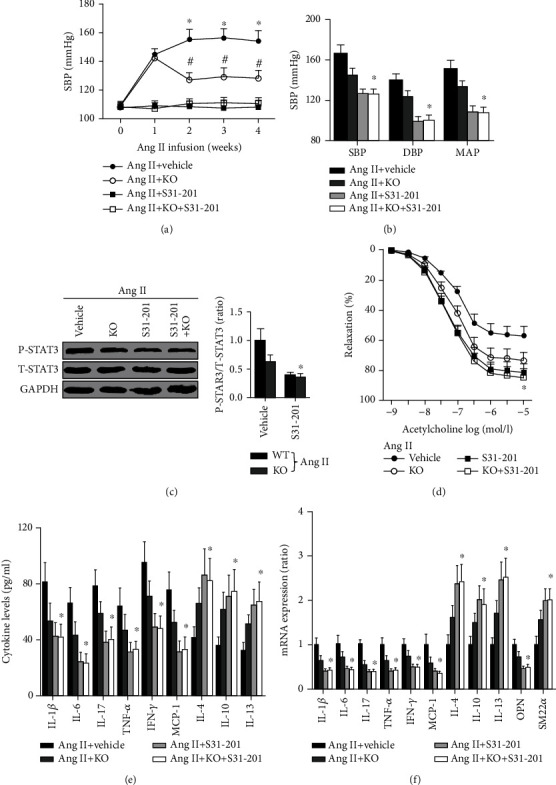
Effects of S31-201 on blood pressure, inflammation, and phenotypic transformation of smooth muscle. (a, b) Blood pressure was determined using the tail-cuff method and the Millar Pressure Volume System; *N* = 10 in each group. (c) The STAT3 phosphorylation in each group was measured. (d) The vascular function for the four groups was detected. (e) Serum cytokine levels were measured using ELISA kits. (f) Aortic mRNA expression of cytokines was analyzed by RT-PCR. *N* = 5 in each group; ^∗^*p* < 0.05 vs. the IL-9-/- Ang II+DMSO group.
